# Lenalidomide decreased the PSA level for castration‐resistant prostate cancer: a case report

**DOI:** 10.1002/ccr3.1328

**Published:** 2018-01-11

**Authors:** Kota Shimokihara, Takashi Kawahara, Taisei Suzuki, Taku Mochizuki, Daiji Takamoto, Jun‐ichi Teranishi, Yasuhide Miyoshi, Yasushi Yumura, Masahiro Yao, Hiroji Uemura

**Affiliations:** ^1^ Departments of Urology and Renal Transplantation Yokohama City University Medical Center Yokohama Japan; ^2^ Department of Hematology Yokohama City University Medical Center Yokohama Japan; ^3^ Department of Urology Yokohama City University Graduate School of Medicine Yokohama Japan

**Keywords:** Castration‐resistant prostate cancer, lenalidomide, thalidomide

## Abstract

Lenalidomide has been developed as the derivative of thalidomide that has fewer side effects. We herein report a rare case of castration‐resistant prostate cancer successfully maintained using lenalidomide for multiple myeloma.

## Introduction

Prostate cancer is the most common cancer among men, and its incidence has markedly increased in recent years 1. Androgen deprivation therapy (ADT) is the standard treatment for advanced or metastatic prostate cancer. However, despite an initially high response rate to ADT, most patients eventually develop castration‐resistant prostate cancer (CRPC) 2, 3, 4, 5. Several treatments have been shown to prolong the overall survival in patients with metastatic CRPC (mCRPC). We herein report a rare case of CRPC successfully controlled with lenalidomide.

## Case Presentation

**Figure 1:**
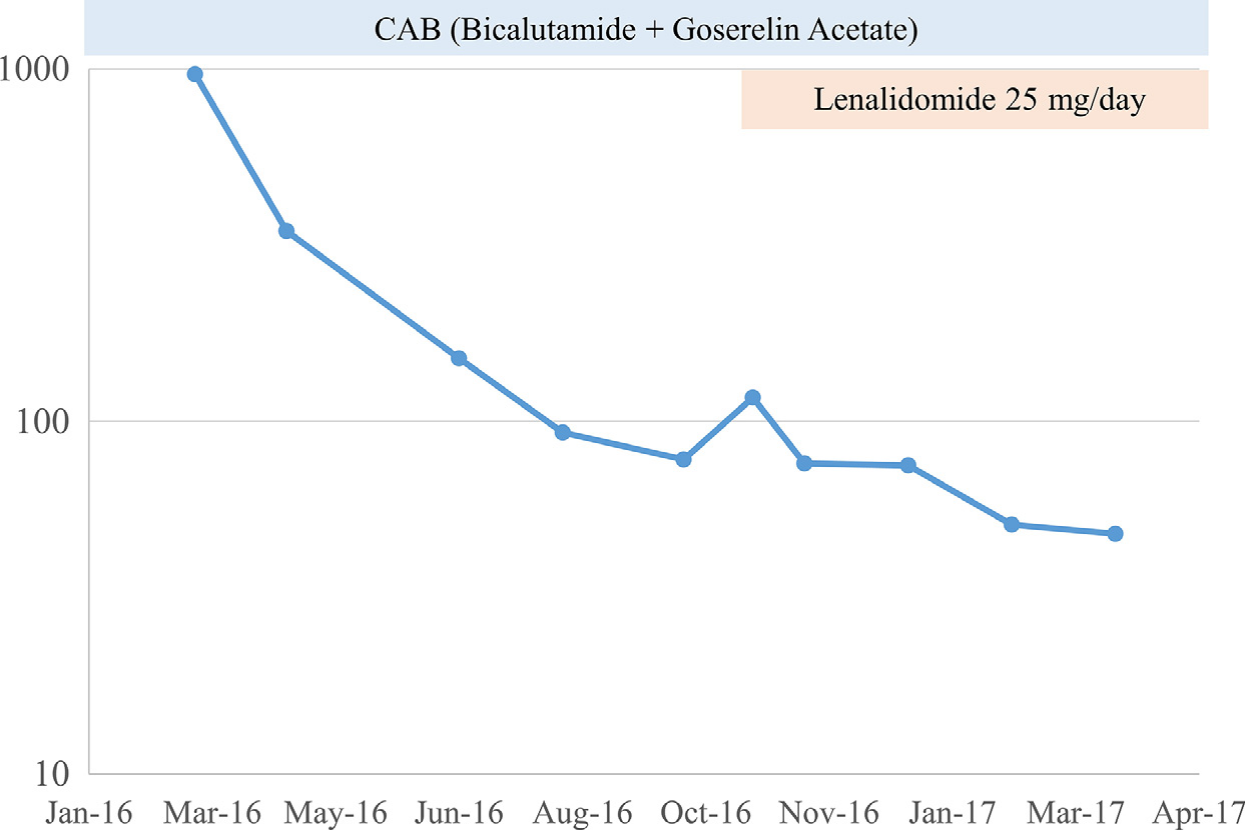
PSA changes.

**Figure 2:**
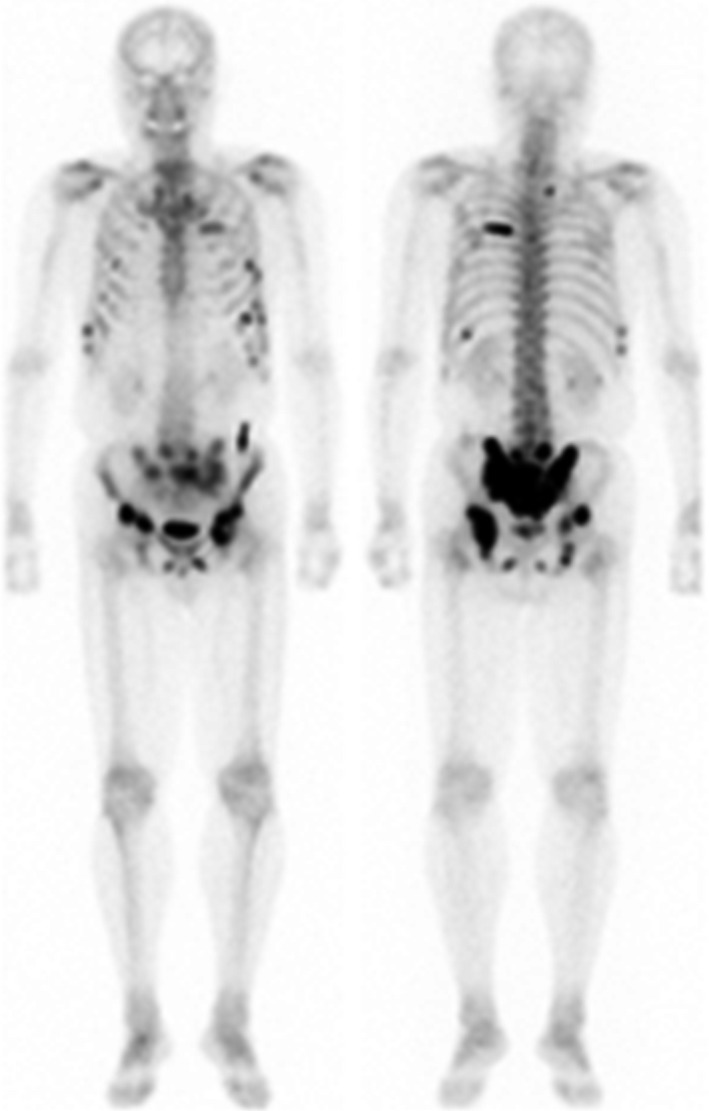
Bone scintigraphy at the time of the diagnosis. Bone scintigraphy showed multiple bone metastasis at pelvis, ribs, and vertebra.

**Figure 3:**
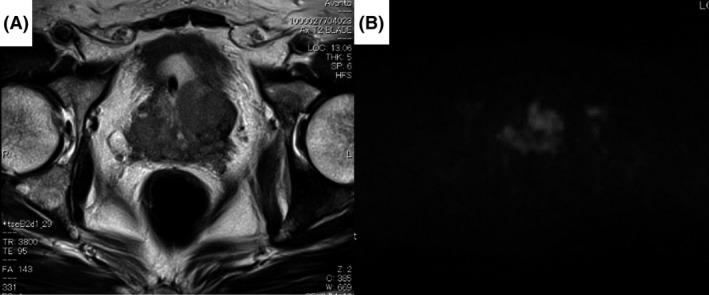
MRI (A) T2WI and (B) diffusion‐weighted image. MRI showed T2 low and DWI high‐intensity area at bilateral lobes of prostate and seminal vesicle. It suggested that prostate cancer infiltrated seminal vesicle.

A 70‐year‐old Japanese man was referred to our hospital for the further examination of his urine retention. On his initial visit, his PSA level was 966 ng/mL, and a prostate needle biopsy was performed. Based on the diagnosis of Gleason score 4 + 4 = 8 prostate adenocarcinoma, bone scintigraphy, CT, and MRI, we diagnosed that his prostate cancer stage was T3bN0M1b. Combined androgen blockade therapy (CAB) using bicalutamide and leuprorelin acetate was started from May 2016 [Figs. 1, 2, and 3]. At his initial examination, because a discrepancy between the total protein (12.3 mg/dL) and albumin (3.6 mg/dL) was observed, multiple myeloma was also suspected. His serum IgG level was elevated to 7126 mg/dL, and a bone marrow biopsy revealed multiple myeloma (IgG‐λ type). Despite an initial response, his PSA level rose again in August 2016. At the same time, because his multiple myeloma was also refractory to the initial chemotherapy of bortezomib, melphalan, and prednisolone (VMP), lenalidomide was introduced. His PSA level subsequently gradually decreased and remained low without any change in the CAB [Fig. 2].

## Discussion

PC is the most common malignancy among men in the Western world 1. In Japan, the morbidity rate of PC has increased rapidly and will be the second‐most common disease after lung cancer in 2020. While advanced and metastatic PC is generally treated with ADT, which is effective, PC eventually progressed to CRPC within a few years. Despite the introduction of new agents, including enzalutamide, abiraterone acetate, docetaxel, cabazitaxel, and Ra‐223, the outcome has not been satisfactory.

In the 1960s, thalidomide was introduced as a sleeping pill. Due to its potential teratogenic effects, this drug was prohibited in 1962. However, in the 1990s, thalidomide was re‐introduced as a neovascularization inhibitor for multiple myeloma 6. The antitumor mechanism of thalidomide involves the suppression of COX‐2 and production of TNF‐α 7, 8.

Recently, thalidomide has attracted attention for its application to PC therapy. In CRPC, thalidomide monotherapy has shown 15%–18% efficacy 9, 10. Furthermore, thalidomide in combination with docetaxel induced a PSA response (more than 50% decrease) in 53% of patients 11. Thalidomide was also reported to be effective in combination with granulocyte–macrophage colony‐stimulating factor or with docetaxel and bevacizumab 12, 13.

Reported toxicities associated with thalidomide include peripheral neuropathy, digestive symptom, psychoneurotic symptom, and deep vein thrombosis, among others. Thalidomide‐induced thrombosis is reported to occur at a rate of 3% in thalidomide monotherapy and 19% in combination with docetaxel. In these case, heparinization is applied and has worked well for to protect against thalidomide‐induced thrombosis 14. Lenalidomide was later developed to decrease these side effects and is now widely used in clinical settings.

However, in 2015, Petrylak et al. 15 reported that lenalidomide with docetaxel therapy resulted in a poorer prognosis than docetaxel with a placebo group in a phase III study, resulting in this study being stopped. In the present case, the PSA level responded and was maintained by lenalidomide administered to control multiple myeloma. In May 2017, the patient's PSA level was still well‐controlled without toxicity.

At present, the efficacy and safety of lenalidomide have not been established. However, as in the present case, some patients with CRPC may benefit from lenalidomide.

## Conclusion

We herein reported a rare case of CRPC successfully maintained with lenalidomide for multiple myeloma.

## Consent for Publication

Written informed consent was obtained from the patients. A copy of the written consent forms is available for review from the Editor‐in‐Chief of this journal.

## Availability of Data and Material

Due to ethical restrictions, the raw data underlying in this article are available upon request to the corresponding author.

## Competing Interests

We declare no conflicts of interest.

## Authorship

KS, TK, and HU: conceived and designed the experiments. KS, TK, and HU: analyzed data. TK, TS, YH, ST, TM, DT, YH, JT, YM, and YY: performed the experiments. KS, TK and MY: wrote the manuscript.
